# Value of preoperative ultrasound in evaluating the peritoneal cancer index of pseudomyxoma peritonei

**DOI:** 10.1186/s12957-019-1730-5

**Published:** 2019-11-12

**Authors:** Lei Liang, Wenhai Wang, Nan Zhou, Jun Guo, Yiyan Lu, Hongbin Xu, Shutian Zhang

**Affiliations:** 1 Department of Gastroenterology, Beijing Friendship Hospital, Capital Medical University, Beijing, China; 2 Department of Ultrasound, Aerospace Central Hospital, Beijing, China; 3 Department of Pathology, Aerospace Central Hospital, Beijing, China; 4 Department of Surgery, Aerospace Central Hospital, Beijing, China; 5 National Clinical Research Center for Digestive Diseases, Beijing Digestive Disease Center, Beijing Key Laboratory for Precancerous Lesion of Digestive Diseases, Beijing, China

**Keywords:** Peritoneal cancer index, Pseudomyxoma peritonei, Ultrasound examination

## Abstract

**Purpose:**

This study aimed to explore the value of preoperative ultrasonography (US) in evaluating the peritoneal cancer index (PCI) of pseudomyxoma peritonei (PMP).

**Methods:**

An ultrasound examination was performed on 59 patients with PMP before surgery, and the ultrasound PCI was evaluated. The accuracy of ultrasound PCI score was evaluated with the surgical PCI score as the gold standard.

**Results:**

The preoperative ultrasound PCI was compared with the surgical PCI. The Spearman correlation coefficient of the total PCI score was 0.608 (*P* < 0.05). The difference in the Spearman correlation coefficient between the preoperative ultrasound PCI and the surgical PCI in areas 0–7 was statistically significant. (1) Among them, the total score and the correlation between 0–3 and 6 were higher. (2) Compared with the surgical PCI, overestimation (> 20%) was concentrated mainly in areas 2 and 4–8 for 2 points, and underestimation (< 20%) was concentrated mainly in areas 1, 3, 4, and 8 for 3 points. (3) The sensitivity and specificity of preoperative ultrasound for predicting the presence or absence of lesions were 85.7% and 50.0%, respectively. The sensitivity of LS 1, LS 2, and LS 3 was 31.7%, 48.2%, and 71.0%, respectively, and the specificity was 44.8%, 55.3%, and 58.8%, respectively.

**Conclusion:**

The ultrasound examination can be used to score the preoperative PCI, judge the severity, and predict the prognosis in patients with PMP.

## Introduction

Pseudomyxoma peritonei (PMP) is characterized by a large number of mucous jelly-like substances dispersed in the peritoneum or omentum, which is rare in clinical practice. The annual incidence of PMP is only one million [1], and the most common position is the vermiform appendix (> 90%). It can also be found in other organs such as the colorectum, gallbladder, stomach, pancreas, ovary, and urachus [2–5]. The main characteristic of this disease is that mucous exocrine cells are diffusely implanted in the omentum, peritoneum, and abdominal organs. Due to the lack of specific clinical manifestations and examination methods, early peritoneal pseudomyxoma is rarely diagnosed before surgery, which makes it extremely difficult to treat the disease. If no active treatment measures are taken, the mean survival time of patients is only 6–8 months [6]. Once such patients are found, cytoreduction surgery (CRS) combined with hyperthermic perioperative chemotherapy (HIPEC) is often used as a treatment option to achieve long-term survival and improve the prognosis of patients [7]. Peritoneal cancer index (PCI) represents the degree of tumor spread in the abdominal cavity. Accurate assessment of PCI in patients with PMP before surgery determines the extent of complete removal of abdominal pelvic tumors during surgery and has an important impact on patient survival. Therefore, preoperative evaluation of the extent of lesion dissemination is important to determine the prognosis of patients. Currently, CT is commonly used to evaluate the degree of peritoneal tumor spread. However, CT is of limited value in the diagnosis of small mucinous tumors, and the evaluation value of CT for preoperative PCI is limited. Chua’s study [8] showed that CT easily underestimates surgical PCI. Ultrasound is simple, inexpensive, and can well distinguish cystic and solid lesions in the abdominal cavity. Moreover, a few reports are available on the application of preoperative ultrasonography in predicting the PCI score and the analysis of its accuracy mechanism. Therefore, the purpose of this study was to predict the PCI by performing preoperative ultrasonography so as to better judge the severity of peritoneal disease and predict the prognosis of patients.

## Materials and methods

### Participants

Fifty-nine patients with PMP treated with CRS + HIPEC in the hospital from April 2017 to May 2018 were selected. Ultrasonography was performed in the department before surgery. The PCI score and pathological results were obtained after surgery. Patients who had multiple operations and could not obtain surgical scores were excluded. All included patients signed informed consent. This study was approved by the Hospital Ethics Committee (No. 20161228-YN-09).

### Instruments and methods

The participants were examined by ultrasound 2 weeks before the surgery. Philip iU22 color Doppler ultrasound diagnostic instrument (C5-3 convex array probe, probe frequency 3–5 MHz) and SuperSonic Aixplorer color ultrasound diagnostic instrument (SC6-1 convex array probe, probe frequency 1–6 MHz) were used to scan the abdominal cavity of patients by two ultrasound doctors engaged in ultrasound diagnosis for more than 5 years, detect lesions, if any, in the corresponding regions of abdominal cavity, and measure lesion size. Ultrasound PCI scoring was carried out during scanning. Two experienced ultrasound doctors observed the results of ultrasonography at the same time and reached a unified conclusion after discussion and consultation on the controversial cases.

### Scoring methods

**Fig. 1:**
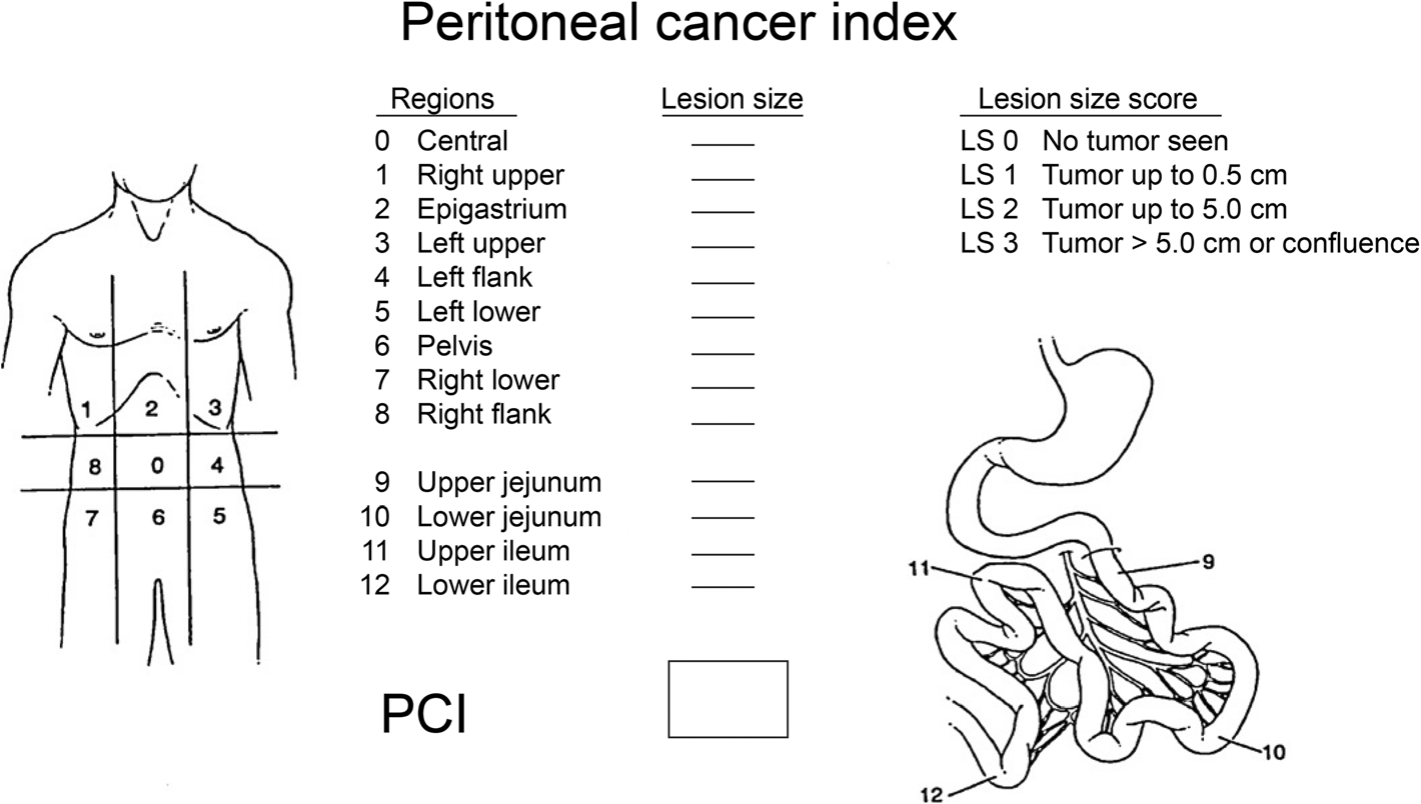
Sugarbaker’s peritoneal cancer index

Ultrasound PCI scoring was done according to the principle of surgical PCI score [9]. The abdominal and pelvic cavity was divided into 13 regions, and each region was given a score 0–3 points according to the lesion size (LS): LS 0, no tumor seen; LS 1, tumor up to 0.5 cm; LS 2, tumor up to 5.0 cm; and LS 3, tumor > 5.0 cm. The score of each region was calculated, and the PCI score was obtained by adding them together (Fig. 1). However, in this study, four regions of the small intestine (regions 9–12) were excluded from the calculation of ultrasound PCI and surgical PCI because the effect of ultrasound on these regions was not good. Therefore, in this study, the ultrasound PCI score and the surgical PCI score ranged from 0 to 27 points. Finally, the participants underwent surgical treatment. The surgical PCI was evaluated during the surgery and recorded after the surgery.

R0: Middle abdomen, including the greater omentum and transverse colon;

R1: Right upper, including the right upper liver, right inferior diaphragmatic surface, and upper posterior surface of the right liver;

R2: Epigastrium, including the left hepatic lobe, lesser omentum, falciform ligament, and upper abdominal fat pad;

R3: Left upper, including the spleen, tail of pancreas, stomach, and left inferior diaphragmatic surface;

R4: Left flank, including the descending colon and left ventral groove;

R5: Left lower, including the sigmoid colon and lateral wall of the left pelvis;

R6: Pelvis, including the female internal genital organs, bladder, sigmoid colon, and Douglas bag;

R7: Right lower, including the cecum, vermiform appendix, and lateral wall of the right pelvis;

R8: Right flank, including the ascending colon and right abdominal cavity;

R9: Upper jejunum;

R10: Lower jejunum;

R11: Upper ileum;

R12: Lower ileum.

### Statistical analysis

The SPSS24.0 software was used for statistical analysis. Enumeration data were expressed as frequency. Measurement data were expressed as mean ± standard deviation and tested using the *t* test. Spearman’s correlation analysis was used to compare the correlation between the preoperative ultrasound PCI score and the surgical PCI score. Sensitivity analysis was carried out for the accuracy of preoperative ultrasonography in detecting tumors with different sizes. A *P* value less than 0.05 was considered as a significant difference.

## Results

**Fig. 2:**
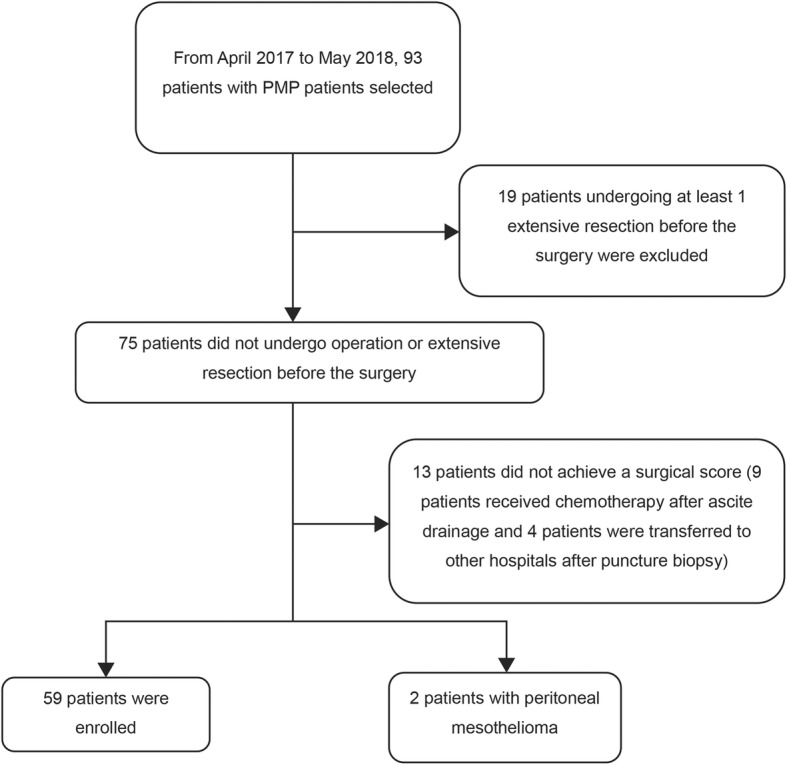
Enrollment flowchart of this study

From April 2017 to May 2018, 93 patients with PMP treated with CRS + HIPEC were admitted to the hospital and underwent preoperative ultrasonography. Further, 19 patients undergoing at least 1 extensive resection before the surgery, 13 patients who did not obtain a surgical score (9 patients received chemotherapy after ascite drainage and 4 patients were transferred to other hospitals after puncture biopsy), and 2 patients with peritoneal mesothelioma were excluded. Finally, 59 patients (31 males and 28 females) were enrolled, and their preoperative ultrasound score and surgical score were obtained (Fig. 2). The average age of the enrolled patients was 55.9 **±** 10.5 years. No significant differences in gender, age, and pathological grade were observed (Table 1).

**Table 1:** Baseline data of enrolled patients in this study

Characteristics	Parameter	Parameter	*P* value
Gender	Male	31	0.795
	Female	28	
Age (year)	Male	54.4 ± 10.3	0.990
	Female	57.6 ± 10.6	
Pathological grade	High	27	0.603
	Low	32	
Origin	Vermiform appendix	50	
	Digestive tract	6	
	Colon	1	
	Cecum	1	
	Others	1	

*P < 0.05: significant difference

### Correlation between preoperative ultrasound PCI and surgical PCI

**Fig. 3:**
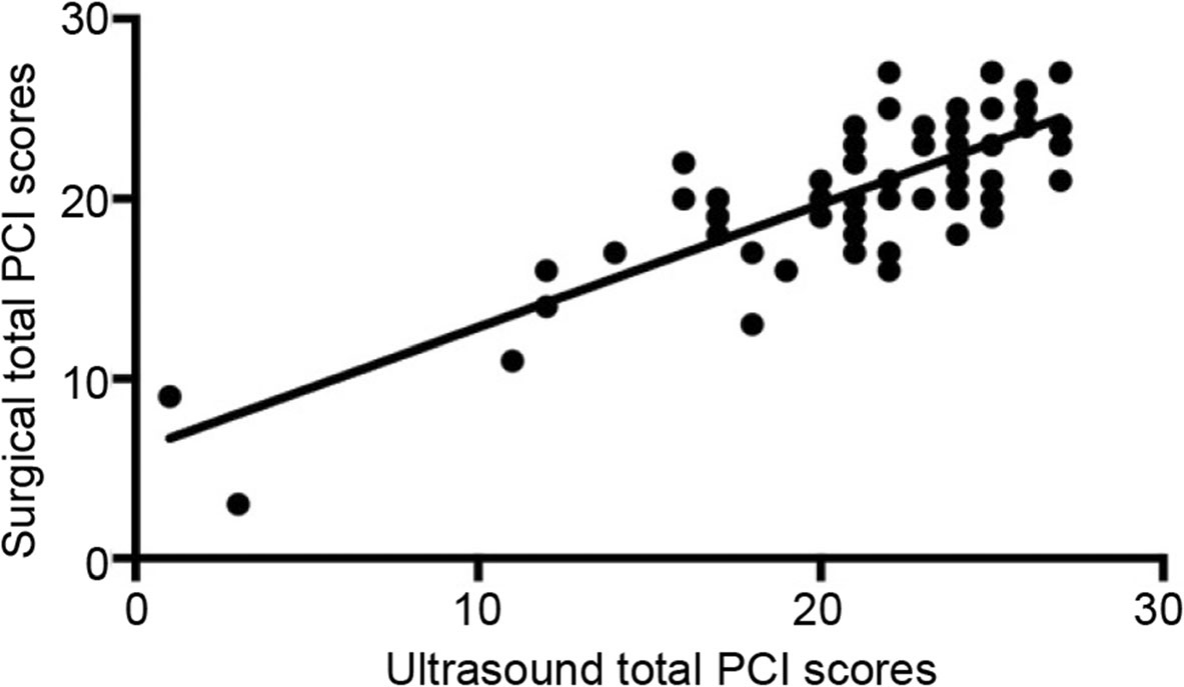
Scatter plot of preoperative ultrasound total PCI score and total surgical PCI score

A monotonic correlation was found between the total PCI score of patients evaluated by preoperative ultrasonography and the total surgical PCI score (Fig. 3). The average of the total PCI score of patients evaluated by preoperative ultrasonography was 20.4 ± 5.0, and the average of the total surgical PCI score was 19.6 ± 4.1 (*P* > 0.05). The Spearman’s correlation coefficient of preoperative ultrasound PCI and surgical PCI was analyzed. The Spearman’s correlation coefficient of total PCI score was 0.608 (*P* < 0.05) and that of regions 0–8 was 0.672, 0.618, 0.589, 0.543, 0.421, 0.370, 0.539, 0.413, and 0.240, respectively. Except for region 8, the differences in Spearman’s correlation coefficient between the preoperative ultrasound PCI score and the surgical PCI score in regions 0–7 were statistically significant. The correlations of total score in regions 0–3 and region 6 were higher (Table 2). Ultrasound images in the right hypochondrium, left hypochondrium, upper abdomen, and pelvic cavity area can be very good to distinguish cystic solid lesions. The diagnostic value of ultrasonography in omentum cake and solid mass of pelvic cavity is higher. However, the evaluation value of ultrasound in the ascending and descending colon, antral colon, and lateral abdominal wall lesions was limited by intestinal gas interference.

**Table 2:** Correlation coefficient between preoperative ultrasound PCI and surgical PCI

Region	Spearman’s correlation coefficient	*P* value	Overestimation rate (%)	Underestimation rate (%)
0	0.672	0.000	3.3	6.8
1	0.618	0.000	15.2	22.0
2	0.589	0.000	22	8.4
3	0.543	0.000	6.7	35.6
4	0.421	0.001	33.8	25.4
5	0.370	0.004	54.2	6.7
6	0.539	0.000	44.0	6.7
7	0.413	0.001	35.6	15.2
8	0.240	0.067	20.0	35.6

### Over and underestimation rates of each region

**Fig. 4:**
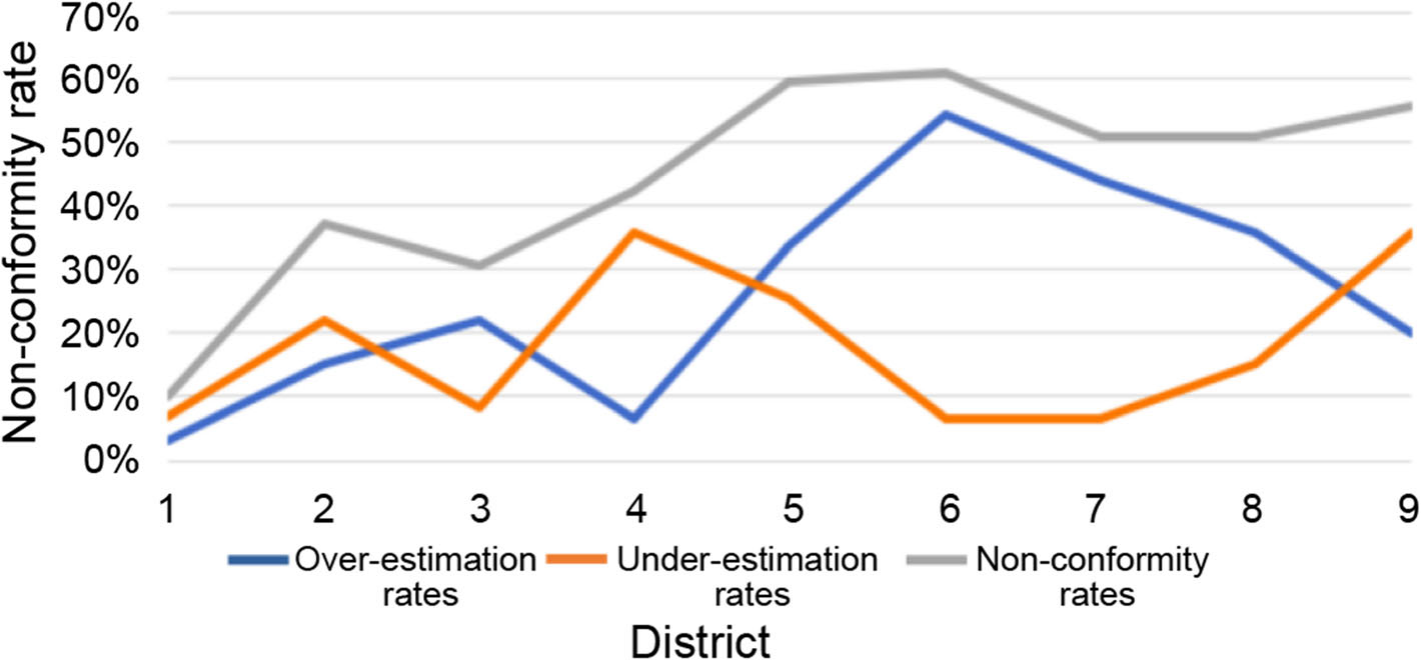
Line chart of under and overestimation rates and nonconformity rates in each region

The overestimation rate of regions 0–8 was 3.3%, 15.2%, 22%, 6.7%, 33.8%, 54.2%, 44.0%, 35.6%, and 20.0%, respectively. The underestimation rate was 6.8%, 22.0%, 8.4%, 35.6%, 25.4%, 6.7%, 6.7%, 15.2%, and 35.6%, respectively (Table 2). The overestimations were concentrated mainly in region 2 and regions 4–8 (> 20%), and the underestimations were concentrated mainly in regions 1, 3, 4, and 8 (> 20%) (Fig. 4). The difference between preoperative PCI and surgical PCI mainly lies in the overestimation of ultrasound score.

### Mechanism of over and underestimating differences in scores of different regions

**Fig. 5:**
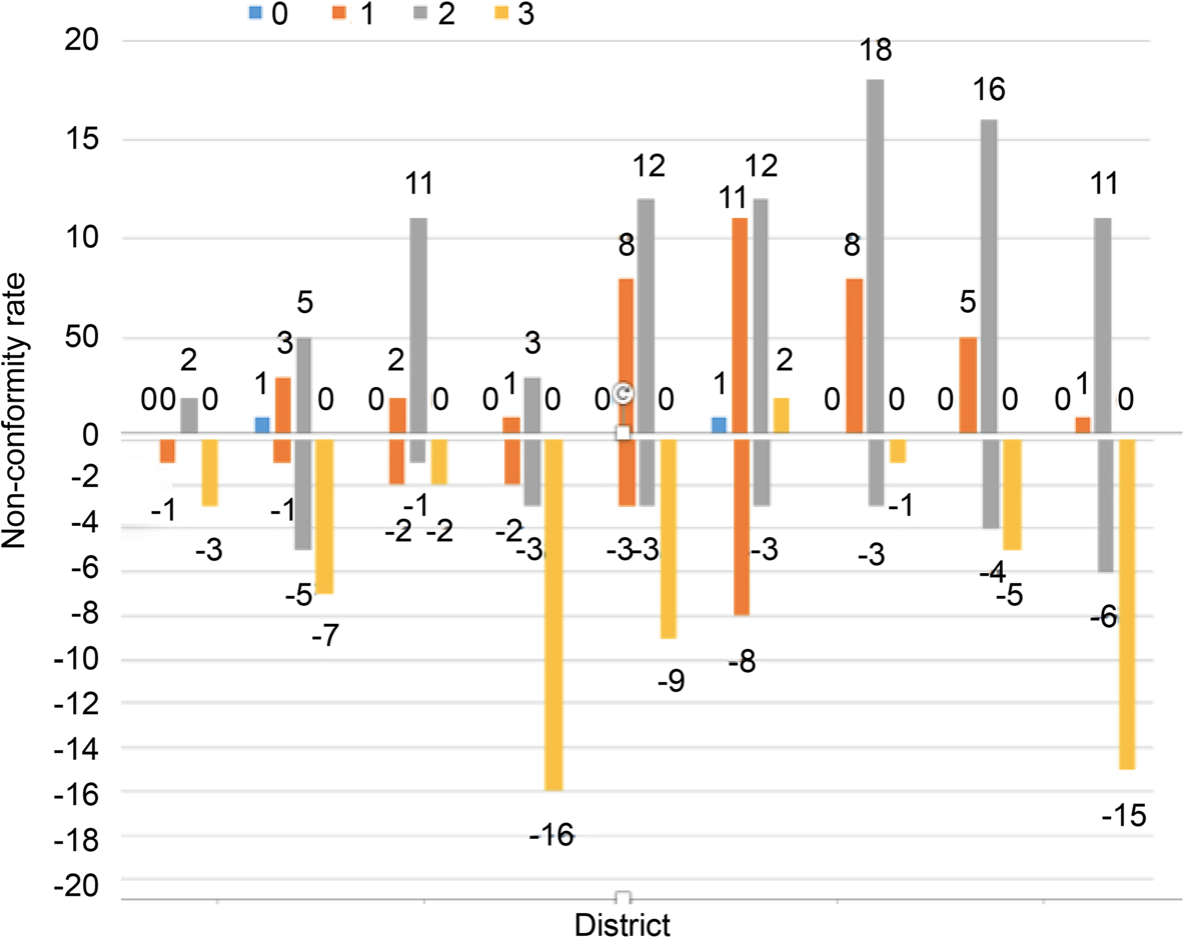
Distribution of over and underestimation of 0–3 points in each region

Region 2 and regions 4–8 were overestimated by 2 points (> 10 patients), regions 3 and 8 were underestimated by 3 points (> 15 patients), region 4 was underestimated by 3 points, and region 1 was underestimated by 2 or 3 points (Fig. 5).

### Sensitivity and specificity of preoperative ultrasound prediction for lesions with different sizes

The sensitivity of LS 0, LS 1, LS 2, and LS 3 was 85.7%, 31.7%, 48.2%, and 71.0%, respectively, and the specificity was 50.0%, 44.8%, 55.3%, and 58.8%, respectively (Table 3). With the increase of lesion size, the sensitivity of preoperative ultrasound to predict lesion size increased from 31.7 to 71.0%, specificity increased from 44.8 to 58.8%.

**Table 3:** Sensitivity and specificity of preoperative ultrasound prediction for different lesion sizes

	LS 0 (%)	LS 1 (%)	LS 2 (%)	LS 3 (%)
Sensitivity	85.7	31.7	48.2	71.0
Specificity	50.0	44.8	55.3	58.8

## Discussion

PMP is characterized by a large amount of gelatinous substances dispersed in the peritoneum or omentum. No specific clinical manifestation is observed in the early stage of the lesion. Most patients visit a doctor because they suffer from abdominal pain and distention caused by the gastrointestinal tract compression due to late lesion, which seriously affects the quality of life of patients. Twenty years ago, Sugarbaker first proposed the combination therapy of tumor reduction and regional chemotherapy for such patients [10]. It has now become the classical treatment for tumorous lesions in the peritoneum. It clears the abdominal lesions to the greatest extent possible and minimizes the volume of residual tumors. At the same time, intraoperative and postoperative intraperitoneal hyperthermic perfusion chemotherapy is used to promote the lethal effect of a local concentration of chemotherapeutic drugs. A multicenter study from 16 centers [11] showed that this combination therapy improved the 10-year survival rate of patients up to 63%. The multivariate analysis showed that the greatest reduction of tumors greatly improved the long-term survival rate of patients. The maximum degree of tumor reduction depended on the distribution and size of the lesion itself, that is, the peritoneal dissemination and implantation of the lesion.

PCI is a quantitative index proposed by Harman and Sugarbaker to describe the extent of peritoneal dissemination of primary or secondary peritoneal neoplasms. It reflects the size and distribution of lesions. This index synthesizes the existing Gilly’s cancer stage, SPCI stage in the Netherlands, and P stage for peritoneal lesions of gastric cancer in Japan. A previous study [11] showed that PCI not only predicted the reduction of tumors in patients but also was independently related to the survival of patients and negatively related to the progression-free survival of patients. Therefore, preoperative evaluation of PCI is particularly important for predicting the severity of disease, choosing clinical treatment methods, and evaluating the prognosis of patients.

Ultrasound and computed tomography (CT) are commonly used in the diagnosis of PMP; magnetic resonance imaging can also be used. CT examination has high spatial resolution and can well display the distribution and morphological features of the lesions, which is of great value in the preoperative diagnosis of PMP. The typical CT manifestations of PMP include [12] a cystic and solid mass in abdominal cavity, scallop-like impression on the visceral infiltration margin of the lesion, thickening of peritoneum and omentum, calcification foci in the abdominal cavity, abdominal effusion with uneven density and small amount of diffuse distribution, enlarged abdominal lymph nodes, small bowel displacement, and so on. Early studies on PMP using CT focused mainly on detecting lesions. However, in recent years, most of them focus on the size of lesions. Based on the specificity of CT in the diagnosis of PMP, the PCI evaluation on PMP is mostly performed using CT in clinical practice. CT has a good correlation with surgical PCI and can be used for preoperative prediction [13]. However, the small tumors in ascites and small septations in masses or thin cyst walls are poorly displayed due to the partial volume effect of CT imaging and the limited resolution of a soft tissue.

Ultrasound can distinguish abdominal cystic and solid lesions well and has a great advantage in diagnosing PMP because it can distinguish the mucous and solid components of the lesions. A previous study reported that PMP had some characteristic manifestations in ultrasonography [14]: cystic and solid masses in abdominal pelvic cavity, heterogeneous echotexture around the liver and spleen, infiltrating lesions of liver and spleen, different degrees of peritoneal omental thickening, myxoedematous ascites (may be accompanied by a floating tumor), and so on. Compared with other imaging examinations, ultrasound has many advantages, such as low cost, real-time dynamics, and greater tolerance among patients. Ultrasound is usually the preferred examination for abdominal lesions. Ultrasound has a potential value in evaluating preoperative PCI based on its remarkable features in diagnosing PMP and differentiating cystic and solid lesions. A few reports are available on the evaluation of PCI using ultrasound. Hence, a definite conclusion on its application value is lacking. A previous study pointed out that the sensitivity and specificity of ultrasound in evaluating PCI were 91.5% and 33.8%, respectively, which were of low value in evaluating the peritoneal dissemination of PMP. However, this study also pointed out that the examiners were the imaging doctors interested in ultrasound, and they were not familiar with standardized scanning in patients with abdominal tumors [15].

In this study, the preoperative ultrasound evaluation of PCI was compared with the surgical evaluation of PCI to explore the application value of ultrasound in the preoperative evaluation of PCI. The results showed that the Spearman’s correlation coefficient between the total score of preoperative ultrasound PCI evaluation and surgical PCI was 0.608 (*P* < 0.05). The preoperative ultrasonography could predict the PCI score. Meanwhile, the correlation coefficient of regions 0–8 was 0.672, 0.618, 0.589, 0.543, 0.421, 0.370, 0.539, 0.413, and 0.240, respectively. Besides region 8, the Spearman’s correlation coefficients between the preoperative ultrasound PCI score and the surgical PCI score in other regions were statistically significant. The preoperative ultrasonography could predict the scores of regions 0–7. Of these, the correlations of the total score in regions 0–3 and 6 were higher. The Spearman’s correlation coefficient between preoperative ultrasound PCI evaluation and surgical PCI in region 0 was higher. The lesions in the greater omentum could be easily detected by ultrasound, and the predictive value of preoperative ultrasonography was good. Region 8 mainly evaluated the ascending colon and the peritoneal lesions around it. The result was inconsistent with the surgical PCI score because of the poor display rate of partial intestinal air shielding. Compared with the descending colon in region 4, the scoring error of the ascending colon in region 8 mainly originated from the overestimation of 2 points and underestimation of 3 points, while the overestimation of 1 and 2 points was concentrated in region 4. Meanwhile, compared with the ascending colon in region 4, the intestinal air of the ascending colon in region 8 was more likely to cause unclear display. Hence, the correlation coefficient in region 8 was not statistically significant, while that in region 4 was statistically significant.

In this study, the reasons for the difference in correlation coefficients of different regions were further analyzed. The over- and underestimation rate in different regions were found to be different. The overestimation rate in regions 0–8 was 3.3%, 15.2%, 22%, 6.7%, 33.8%, 54.2%, 44.0%, 35.6%, and 20.0%, respectively. The underestimation rate was 6.8%, 22.0%, 8.4%, 35.6%, 25.4%, 6.7%, 6.7%, 15.2%, and 35.6%, respectively. Of these, the overestimation rates were concentrated mainly in regions 2 and 4–8 (> 20%), and the underestimation rates were concentrated mainly in regions 1, 3, 4, and 8 (> 20%). Moreover, the distributions of over- and underestimation scores in different regions were analyzed. Region 2 and regions 4–8 were overestimated mainly by 2 points (> 10 patients). For these regions, it was easy for operators to misinterpret the lesions of 0.5–5 cm as those of > 5 cm. The lesions in region 2 were mainly in the perigastric, lesser omental sac, and falciform ligament regions; they were not easily distinguished from the surrounding normal tissues. The lesions in regions 4–8 were mainly in the abdominal and pelvic regions. Most of the patients with mid-late stage cancer in this study had diffuse abdominal lesions. Once diffuse abdominal lesions were found by ultrasound, they were easy to be diagnosed as lesions > 5 cm. Regions 1, 3, 4, and 8 were underestimated mainly by 3 points. The largest lesion size in each region obtained by ultrasound scanning for any section was taken as the scoring basis. The reason might be that the perihepatic and perisplenic lesions of some patients were not fully displayed during scanning, leading to underestimation. The descending colon region might be related to the misjudgment caused by intestinal air shielding. Ultrasound could not distinguish regions 9–12 well because they were the upper jejunum, lower jejunum, upper ileum, and lower ileum. These regions were excluded from the calculation of ultrasound PCI and surgical PCI in this study to avoid bias in the quantification of tumor load in them.

This study showed the sensitivity and specificity of preoperative ultrasound prediction for lesions with different sizes. The sensitivity of LS 0, LS 1, LS 2, and LS 3 was 85.7%, 31.7%, 48.2%, and 71.0%, respectively. The specificity was 50.0%, 44.8%, 55.3%, and 58.8%, respectively. The sensitivity and specificity of ultrasound in predicting lesions were 85.7% and 50.0%, respectively. However, the sensitivity and specificity of ultrasound in predicting tumor load according to Krause [15] were 91.5% and 33.8%, respectively. Compared with this study, the specificity increased in the present study. The sensitivity of preoperative prediction of lesion size by ultrasound increased from 31.7 to 71.0% with the increase in lesion size from LS < 0.5 cm to LS > 5.0 cm, and the specificity increased from 44.8 to 58.8%, which was similar to the results of other imaging methods reported in previous studies [16]. In this study, the sensitivity of preoperative CT prediction for small lesions with LS < 0.5 cm was only 11%. The sensitivity of preoperative CT prediction for lesions with LS > 5 cm was 94% with the increase in lesion volume.

This study had some limitations. Most of the enrolled participants were patients with PMP having extensive dissemination of peritoneal lesions; the PCI was higher, and a few patients had limited early lesions. It was not possible to analyze the preoperative diagnostic efficacy with better prognosis in patients with low-point PCI. Hence, the sample size should be expanded to include early patients in future studies.

In this PMP disease, some tumors are gelatinous nodules, and some tumors are gelatinous material filling the abdomen without a definite border. Our basic criteria in this study of defining a nodule at the site on USG are according to the Sugarbaker classification [17], based on lesion size and distribution. The lesion size of the largest implants is scored (0–3) for each abdominopelvic region. In particular, each region could be assigned zero to three points, with 0 = no lesion identified, 1 = lesion up to 0.5 cm in maximum diameter, 2 = lesion exceeding 0.5 cm but not 5 cm in maximum diameter, and 3 = lesion or confluent lesions exceeding 5 cm in maximum diameter. When the lesion is gelatinous nodules, we can measure the diameter, if the lesion is gelatinous material filling the abdomen without a definite border, it must be more than 5 cm in diameter. According to our study, USG has limitations in determining an accurate extent of tumors in some small bowel regions (regions 9–12). But in abdominopelvic region (region 1–8), we can get a more accurate extent by USG than CT.

For tridimensional tumors, the measurement from diameter alone is limited. A way to classify on the basis of the volume of the gelatinous material would probably be a better way to delineate PCI and a more original method of study. The use of preoperative PCI of patients with PMP is more valid and useful in cases of early disease and possibly in recurrences. In large volume and advanced diseases, a high score would be expected and would not actually justify any change in decision making.

In conclusion, preoperative ultrasonography has a good predictive value for preoperative PCI of patients with PMP. It can evaluate the extent of lesion dissemination and lesion size and also indicate the clinical application before the surgery to a certain extent. It has reference significance for the choice of clinical treatment methods. Ultrasound doctors should carefully evaluate regions easy to be misjudged to avoid over- and underestimation. Early detection of mucous substances is especially important.

## Data Availability

We confirm the availability of data and materials. All the data and materials can be found in the electronic medical records and data system of the Aerospace Central Hospital.

## Abbreviations

PCI: Peritoneal cancer index
PMP: Pseudomyxoma peritonei
US: Ultrasonography
